# Goliath catfish spawning in the far western Amazon confirmed by the distribution of mature adults, drifting larvae and migrating juveniles

**DOI:** 10.1038/srep41784

**Published:** 2017-02-06

**Authors:** Ronaldo B. Barthem, Michael Goulding, Rosseval G. Leite, Carlos Cañas, Bruce Forsberg, Eduardo Venticinque, Paulo Petry, Mauro L. de B. Ribeiro, Junior Chuctaya, Armando Mercado

**Affiliations:** 1 Museu Paraense Emilio Goeldi (Belém, Pará, Brazil), Caixa Postal 399, Belém, 66040-170, PA, Brazil; 2 Wildlife Conservation Society (WCS), 2300 Southern Boulevard, Bronx, 10460, New York, USA; 3 Instituto Nacional de Pesquisas da Amazônia (INPA) Cx Postal 478, Manaus, AM, 69011-970, Brazil; 4 Universidade Federal do Rio Grande do Norte (UFRN), Depto. de Ecologia, 59072-970 - Natal, RN – Brazil; 5 The Nature Conservancy (TNC), 4245 Fairfax Drive, Arlington, VA, 22203 & Museum of Comparative Zoology, Harvard University, MA, Cambridge, USA; 6 Instituto Brasileiro de Geografia e Estatística (IBGE), Reserva Ecológica do IBGE, DF 001, KM 38 - C.P. 8588, Brasília, Brazil; 7 Universidade Federal do Rio Grande do Sul, Post-graduate Programme of Animal Biology, Laboratório de Ictiologia Departamento de Zoologia - UFRGS Av. Bento Gonçalves, 9500 - Bloco IV - Prédio 43435 CEP 91509-900 - Porto Alegre - RS – Brazil

## Abstract

We mapped the inferred long-distance migrations of four species of Amazonian goliath catfishes (*Brachyplatystoma rousseauxii, B. platynemum, B. juruense and B. vaillantii*) based on the presence of individuals with mature gonads and conducted statistical analysis of the expected long-distance downstream migrations of their larvae and juveniles. By linking the distribution of larval, juvenile and mature adult size classes across the Amazon, the results showed: (i) that the main spawning regions of these goliath catfish species are in the western Amazon; (ii) at least three species—*B. rousseauxii, B. platynemum, and B. juruense*—spawn partially or mainly as far upstream as the Andes; (iii) the main spawning area of *B. rousseauxii* is in or near the Andes; and (iv) the life history migration distances of *B. rousseauxii* are the longest strictly freshwater fish migrations in the world. These results provide an empirical baseline for tagging experiments, life histories extrapolated from otolith microchemistry interpretations and other methods to establish goliath catfish migratory routes, their seasonal timing and possible return (homing) to western headwater tributaries where they were born.

The Amazon has two main groups of migratory fish species, and they belong to the orders Siluriformes (catfishes) and Characiformes (characins)^1^,^2^,^3^. Major fisheries in the Amazon are based on knowledge of the seasonal upstream and downstream movements of fish, although the life cycles are poorly known by fishermen because the species enter and leave local fishing areas^4^,^5^,^6^. Long-distance fish migrations (>1,000 km) that exclusively or partially involve freshwater are known for salmon^7^ and eels^8^ but have also been inferred for Amazon goliath catfishes of the family Pimelodidae^9^,^10^,^11^,^12^. Although the migrations of most catfish species are poorly known, the general pattern reported is upstream movement to spawn, downstream passive drifting and even active migration of young juveniles to enter nursery habitats in river channels, floodplains or estuaries^7^,^13^,^14^,^15^. In the Amazon, goliath catfishes are major river channel and estuarine predators that are represented by a paraphyletic group of six extant and one fossil species of the genus *Brachyplatystoma*^16^,^17^,^18^ with a maximum known adult fork length (FL) of 60–280 cm^9^ (Fig. 1).

The first hypotheses of goliath catfish migration focused on *B. rousseauxii* and *B. vaillantii*, the most important commercial species, and their dependence on the Amazon River estuary as their nursery and inland rivers as feeding and spawning areas^9^. The spawning areas, however, were only identified as being located in the western Amazon, a vast region of at least 2 million km^2^ that includes parts of Colombia, Ecuador, Peru, Bolivia and Brazil. More recent biological studies based on commercial fisheries in all major western Andes-Amazon tributary basins^19^,^20^,^21^,^22^,^23^ established the seasonal, and in some cases year-round, presence of mature goliath catfishes in the western Amazon to at least a few hundred km downstream of the Andes. However, these investigations could not verify the spawning areas, leaving the possibility that they are located farther upriver and closer to the Andes.

We present data on the distribution of goliath catfish (*Brachyplatystoma*) adults, larvae and juveniles across the Amazon Basin, including areas in or near the Andes. Even without tagging experiments, the general differential distribution of sub-adult (downstream) and adult (upstream) goliath catfish size-classes^9^, as well as otolith microchemistry data^10^, strongly indicates that long-distance upstream goliath catfish migration occurs. Seasonal upstream goliath catfish movements are also visually obvious at some cataracts, such as those of the Madeira River (Fig. 1) in Brazil, before dams were built^4^, and those of the Caquetá River in Colombia^6^. Therefore, it is reasonable to hypothesize that long-distance downstream migrations of young fish occurs, otherwise there would be no recruitment to nurseries, in some cases as far downstream as the Amazon River estuary.

To test the goliath catfish migratory hypothesis related to western spawning regions, we mapped the presence of mature adults complemented by the seasonal and geographical variation in abundance and length of larvae and juveniles in river channels across the Amazon, including in the estuary. Two complementary geographical and temporal perspectives were used. The first focused on the Madeira Basin, the Amazon’s largest sub-basin with headwaters in the Andes, and where year-round collections were performed. The second included most of the Amazon and available data from all years. Our study shows that spawning for at least *B. rousseauxii, B. juruense* and *B. platynemum* occurs in or near the Andes and demonstrates conclusively that long-distance downstream migration of their larvae and juveniles occurs. The great variation in *B. platynemum* larvae size across the Amazon indicates a much wider nursery area than just the Andean region. Size-class data for *B. vaillantii* indicate long-distance migration and spawning in the western Amazon but do not confirm it near the Andes. Our results are also discussed in light of published goliath life history hypotheses derived from genetic and otolith isotope signatures.

## Results

### Adult Distribution and Gonadal Stage

**Figure 2:**
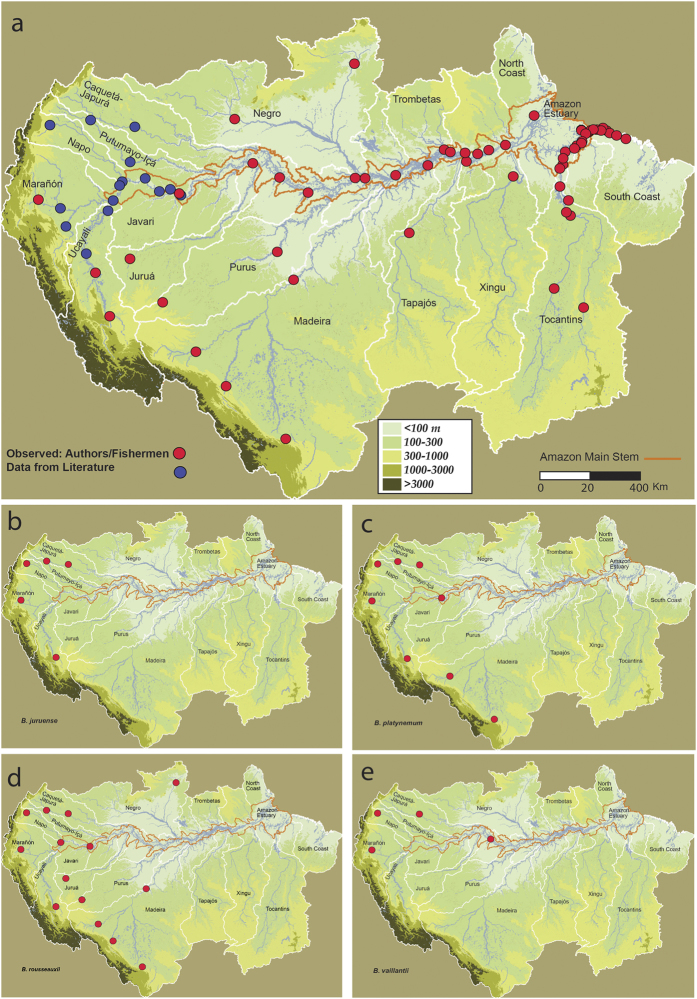
Distribution of sites investigated and locations of mature goliath catfishes. (**a**) Sites investigated by the authors and others from published data. (**b**–**e**) Locations of mature goliath catfishes by species. Figure was created by authors with ArcGIS for Desktop Advanced 10.2, MapPublisher 9.6 tool inside Adobe Illustrator CC, and Adobe Illustrator CC, 2.0.

Fish length and gonadal data derived from specimens captured in fisheries in major Andes-Amazon sub-basins in Brazil, Bolivia and Peru, complemented by published data from studies based in Colombia^19^,^23^ and Ecuador^24^, show the wide distribution of mature *B. rousseauxii, B. platynemum* and *B. juruense* in all major turbid rivers with headwaters in the Andes, including the Amazon River main channel (Fig. 2 and Table 1). Mature fish are defined as ripe individuals, that is, individuals with fully developed ova or testes^25^. *Brachyplatystoma rousseauxii* has the widest distribution. In addition to whitewater (turbid) rivers arising in the Andes, it is found in many clearwater and blackwater tributaries that arise on the Brazilian and Guiana Shields. With the exception of the lower Tocantins River^26^, which is part of the Amazon River estuary, *B. rousseauxii* is rarely registered in the fisheries of blackwater and clearwater rivers, an indication of its rarity in these drainages. The only Brazilian or Guiana shield rivers where mature long-distance migratory goliath catfishes were found was the Branco, a semi-turbid tributary of the Negro River. Of the goliath species considered, only *B. rousseauxii* was present in the Branco River, but it is of minimal importance in fisheries there and is reported by fishermen to be relatively rare.

**Figure 3:**
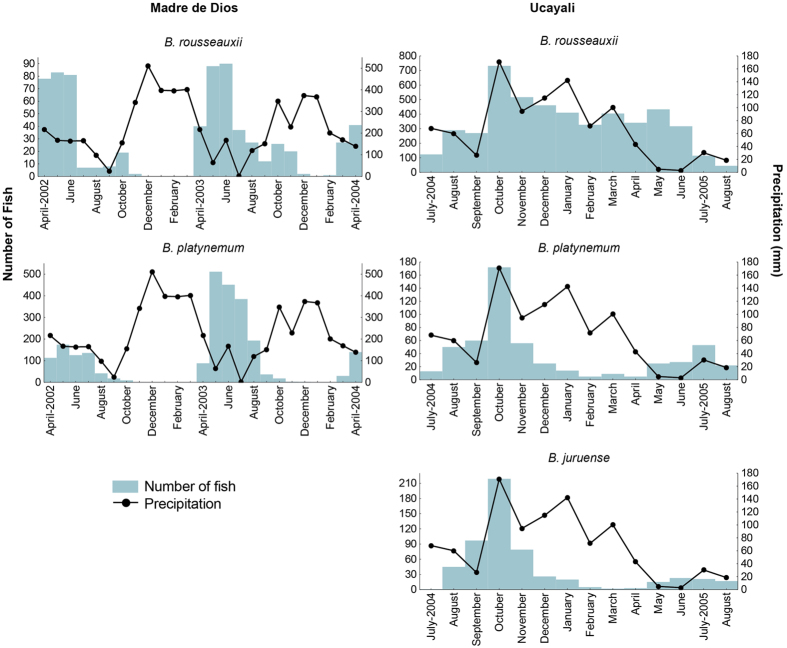
Mature goliath catfish from commercial catches near the Andes indicate the presence of spawning populations. Monthly precipitation and monthly capture of mature *B. rousseauxii, B. platynemum*, and *B. juruense* between April 2002 and April 2004 in Puerto Maldonado (Madre de Dios River, Upper Madeira Basin) and between July 2004 and July 2005 at Atalaya (near the confluence of the Ucayali and Urubamba Rivers). *B. juruense* was not captured in the Madre de Dios River during our study period. Precipitation was used as a proxy for river level since there were few data for the latter in the Andean region.

In contrast to the other three widely distributed species discussed in this paper, *B. vaillantii* is rare or naturally absent above the Madeira Rapids in the southwestern Amazon of Bolivia and Peru. The new fishways around the Madeira dams, however, could allow *B. vaillantii* to migrate more easily to Bolivian and Peruvian waters in the southwestern Amazon, which would alter the population dynamics of this species and perhaps other species on which it preys. Mature *B. rousseauxii, B. platynemum* and *B. juruense* are only abundant, as indicated by fisheries catches, between approximately 55–250 m of elevation and only in turbid rivers at least 3,500 km upstream from the Amazon River mouth (Fig. 2 and Table 1). Considering only adult stages, *B. vaillantii* has the widest distribution of the long-distance migratory goliath catfishes because of its presence in the far eastern Amazon, including the estuary^9^,^27^. In the estuary, however, *B. vaillantii* adults are not mature as defined above. Mature *B. vaillantii* adults are known as far upstream as the Pongo de Manseriche, an Andean gorge of the Marañón River at 246 m elevation in northern Peru and 4,847 km upstream of the Amazon River mouth. Year-round gonadal stage data reported for *B. rousseauxii, B. platynemum* and *B. juruense* captured over a 12-month period by local fishermen in the upper Madeira (2003–2004) and Ucayali (2004–2005) basins of Peru provided unequivocal proof of the presence of only mature goliath catfishes in or near the Andes to at least 198 m elevation and 5,788 km from the Amazon River mouth (Figs 2 and 3 and Table 2). Mature *B. rousseauxii* and *B. platynemum* were abundant in commercial fisheries in both areas during our sampling periods, whereas *B. juruense* was only captured in the Ucayali River. Other goliath catfishes (*B. filamentosum, B. capapretum*, and *B. tigrinum*) are also present in these rivers but in smaller quantities, as indicated by commercial fisheries^28^,^29^.

All 5,348 *B. rousseauxii*, 2,985 *B. platynemum* and 528 *B. juruense* specimens we examined from commercial fisheries near the Andean Piedmont or in the immediate pre-Andean area were sexually mature adults with fully developed gonads or recently spawned fish (Table 2). In contrast to the downstream regions, all goliath catfish captured near the Andean Piedmont had empty stomachs, suggesting that their presence in these areas was not related to trophic migration but to spawning. Although goliath catfishes were present in every month in the far western Amazon, the commercial captures from which our data were derived indicated strong seasonal variation (Fig. 3). The seasonal differences between commercial captures in the Madre de Dios (upper Madeira Basin) and Ucayali and Urubamba Rivers (Ucayali Basin) (Fig. 3) may reflect distinct headwater migration patterns or differences in fishing, considering that fishing was virtually halted in the Madre de Dios during the high water discharge period (November-February) due to the large quantities of downstream-moving wood that are a danger to nets.

In the Ucayali and Urubamba Rivers, ready-to-spawn *B. rousseauxii, B. juruense* and *B. platynemum* were most abundant in commercial fisheries during the rainy and warmer months corresponding to the higher river discharge period from October to March (Fig. 3). Since monthly fishing effort was approximately equivalent, landings can be considered a proxy for seasonal migratory fish abundance. Furthermore, goliath catfishes were captured by commercial fisheries in river channels with downriver drifting gill nets during all months. Since drifting gill nets only capture fish moving upstream, they reveal the direction of movement.

### Seasonal Larval Densities as Spawning Indicators

**Figure 4:**
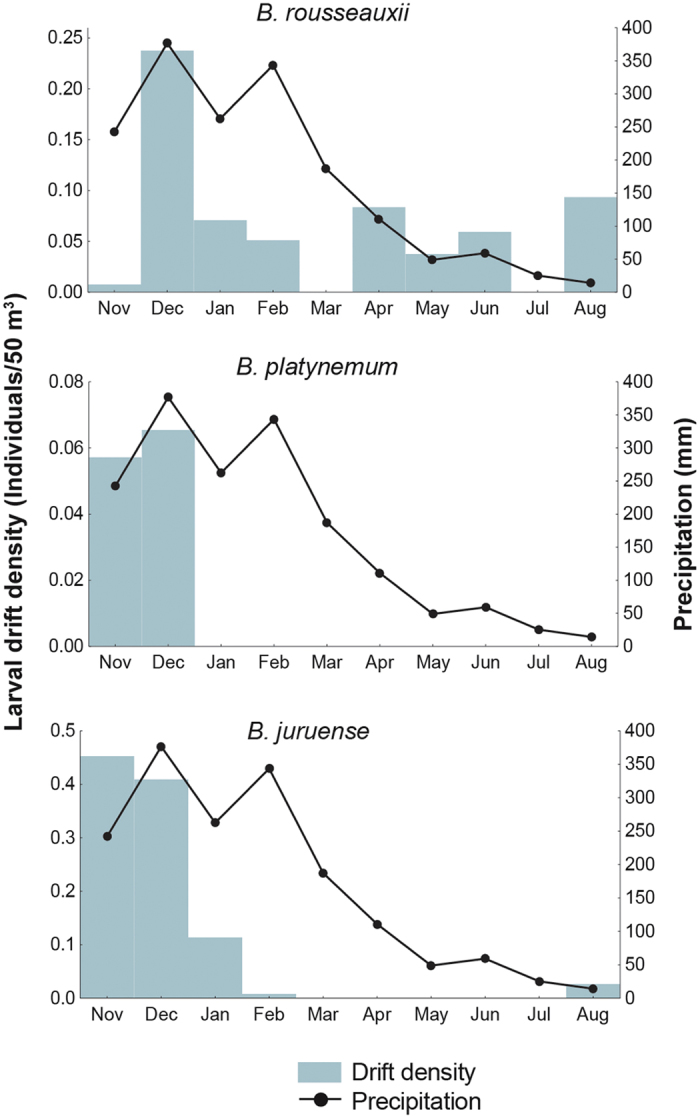
Goliath catfish larvae densities based on standardized sampling as indicators of spawning periods. The monthly drifting larvae densities of goliath catfishes in the Madre de Dios River of the upper Madeira Basin in relation to total monthly precipitation (mm) levels. Larvae densities were estimated based on ichthyoplankton samples from the Madre de Dios River between November 2004 and August 2005. Precipitation data were used as a proxy for river level since data for the latter were not available. Precipitation data are for the city of Puerto Maldonado were supplied by the Servicio Nacional de Meteorología e Hidrología del Peru (SENAMHI).

Only *B. rousseauxii* larvae were captured throughout most of the year in standardized density sampling, including during the low water period, but in much lower quantities than during the December floods (Fig. 4). The other two species present in the sampling area, *B. platynemum* and *B. juruense*, were poorly or not represented during 6–8 months in density samples that included parts of the high, low and rising water periods, and only the latter species was captured during the low water period. The highest larval densities of all species combined occurred between November and January, months when river discharge, depth and current velocity were rapidly increasing^30^. Mature *B. platynemum* were also fairly common in Madre de Dios commercial fisheries from May to August, which included the falling and low water period, verifying their presence in the region (Fig. 3). Although *B. juruense* larvae were captured between November 2004 and August 2005, no adults were reported in the commercial fisheries of the Madre de Dios for this period, although the species is known in the study area^31^ (Fig. 3).

### Growth of Larvae and Juveniles

**Figure 5:**
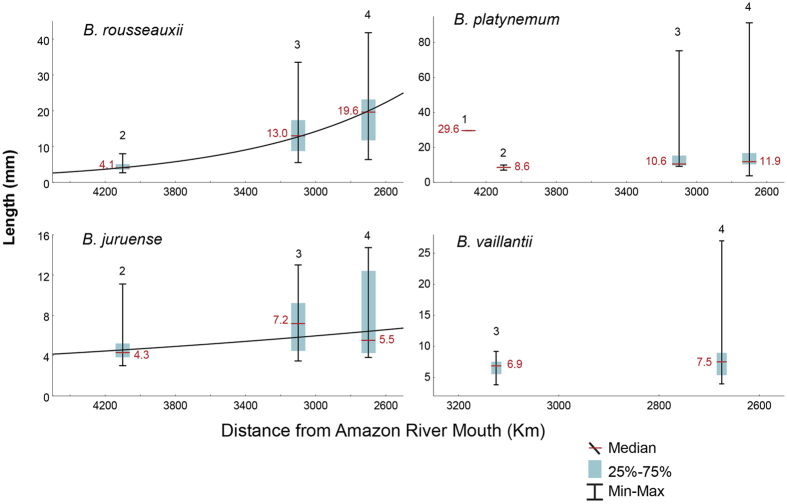
Downriver migration of goliath larvae/juveniles in the Madeira River system. Box-plots of the lengths of goliath catfish drifting larvae from near the Andes in the Madre de Dios River to the Madeira River in Brazil, approximately 1,600 km downriver. Collection sites are indicated by the numbers above the box-plots: 1- Madre de Dios River at Los Amigos River confluence, 2- Madre de Dios River at Puerto Maldonado, 3- Madeira River at Porto Velho, and 4- Madeira River at Humaitá. Data were fit to an exponential curve, where *Lmm* is the size in millimeters and *km* is the distance in kilometers: *B. rousseauxii: Lmm* = 269.1^−0.00101×*km*^, r^2^ = 0.67, F_(1, 327)_ = 670, p < 0.001; *B.* juruense: *Lmm* = 18.5^−0.00034×*km*^, r^2^ = 0.22, F_(1, 76)_ = 23.1, p < 0.001; *B. platynemum*: r^2^ = 0.17 F_(1, 108)_ = 1.93 p > 0.05; *B. vaillantii*: r^2^ = 0.08, F_(1, 34)_ = 2.9, p > 0.05.

**Figure 6:**
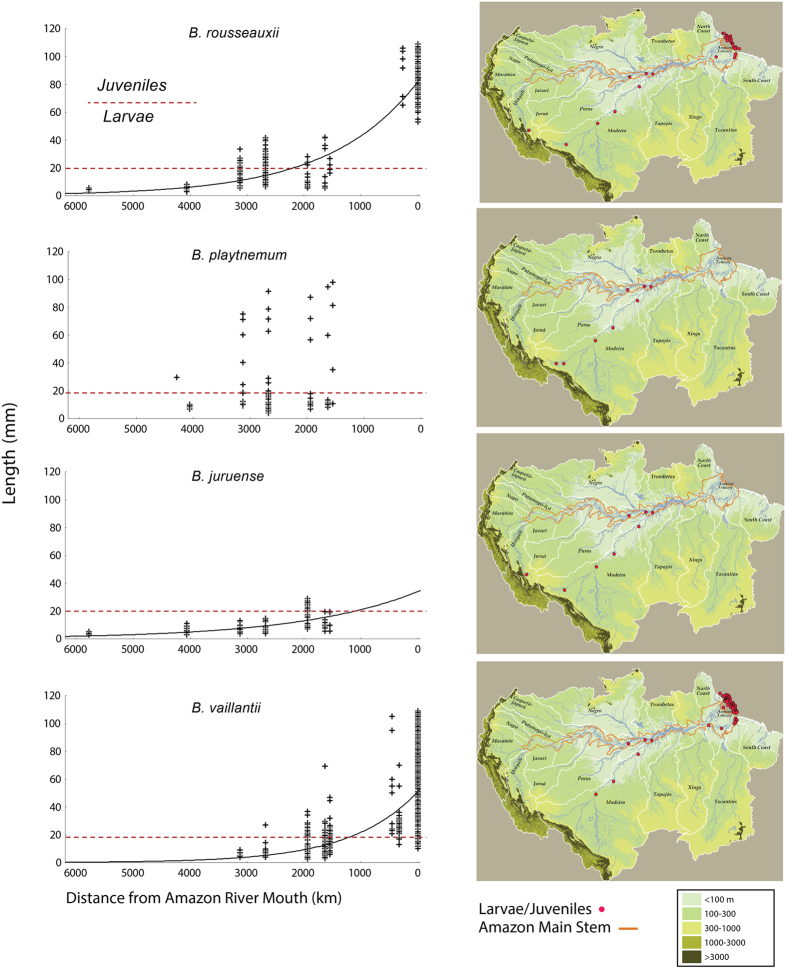
Downriver migration of goliath larvae/juveniles in the Amazon Basin. Fish length scatterplots of goliath catfish drifting larvae from near the Andes to the Amazon River estuary in Brazil. Data were fit to an exponential curve, where *Lmm* is the size in millimeters and *km* is the distance in kilometers: *B. rousseauxii: Lmm* = 81.8^−0.00065×*km*^, r^2^ = 0.84, F_(1, 491)_ = 670, p < 0.001; *B.* juruense: *Lmm* = 33.9^−0.00048×*km*^, r^2^ = 0.57, F_(1, 130)_ = 172.8, p < 0.001; *B. platynemum*: r^2^ = 0.04, F_(1, 151)_ = 6.3, p > 0.05; *B. vaillantii: Lmm* = 50.9^−0.00085×*km*^, r^2^ = 0.64, F_(1, 1035)_ = 1818, p < 0.001. Maps in figure was created by authors with ArcGIS for Desktop Advanced 10.2, MapPublisher 9.6 tool inside Adobe Illustrator CC, and Adobe Illustrator CC, 2.0.

If the goliath catfish spawning areas and nurseries are separated by thousands of kilometers as we hypothesize, it should be reflected in the larvae and juvenile growth in the downstream direction. We tested this hypothesis for the Madeira Basin, for which seasonally sequential data were available, and for the Amazon as whole, including data from various years. The growth of fish in their early phase may be described by Brody’s equation, which relates fish size (L_t_) to time (t) in the exponential equation *L*_*t*_ = *a* × *e*^*K*×*t*^ 32, where *a* and *K* are constants. We used Brody’s equation but substituted the time variable with distance to fit the relationship between larvae/juvenile length and their distance from the mouth of the Amazon River. The size distributions of *B. rousseauxii* and *B. juruense* in the Madeira Basin (Fig. 5) and the Amazon as a whole (Fig. 6) fit exponential curves (p < 0.01 for both species). By the time *B. rousseauxii* enters the Amazon River from the Madeira River, it has reached juvenile size (approximately 20 mm). For *B. juruense*, the available data indicate an increase in median length from the headwaters to the upper Madeira in Brazil, after which the median length decreases, indicating a wide spawning region for the species (Fig. 6). Juvenile stages of *B. juruense* are most common in the central Amazon, approximately 2,000–2,500 km from the Amazon River mouth, though much less is known about this species than the other goliath species considered and more collections are needed in the lower Amazon River, where juveniles are also known.

A seasonal factor for all species is that varying current speeds during sampling periods could affect the larval size distribution along the downstream movement. If current speed greatly influenced the distribution of juvenile size, then a much less precise pattern would be expected for *B. rousseauxii* since it spawns during varying periods of the year with different current speeds. However, *B. rousseauxii* shows the best fit of all species to the downstream migratory growth model.

The wide distribution of small larvae of *B. platynemum* in the Madeira Basin and the Amazon as a whole and their highly mixed length-class distribution do not fit exponential curves, strongly suggesting that spawning for this species is widespread, that more than one population exists^33^ and that the nurseries include a large area in the western and central, and perhaps even eastern, Amazon (Figs 5 and 6).

Larvae and juveniles of *B. vaillantii* have been captured in the lower stretches of the Madeira River and upriver near the Madeira Rapids, at least 3,129 km upstream of the estuary. The presence of small larvae (<5 mm) in this region suggests the proximity of a spawning area, but we have too few sample points to detect the growth of *B. vaillantii* larvae during their downriver migration (Fig. 5). Nevertheless, the data for *B. vaillantii* larvae and juveniles for the entire Amazon fit an exponential curve (p < 0.01), corroborating the long-distance downstream larval and juvenile migration hypothesis (Fig. 6). Given the presence of ripe adults of this species in the Marañón River in Peru at its outlet from the Andes, it is possible that some spawning occurs much farther upstream in the western Amazon than our data indicate. No small *B. vaillantii* larvae (<5 mm) were found in the Amazon River within 1,500 km of the estuary.

## Discussion

We argue that the distribution of mature size classes and general downriver movement, concurrent growth and regional size-class differences of goliath catfish larvae and juveniles of *B. rousseauxii, B. juruense* and *B. vaillantii* indicate that the western spawning areas and downstream nurseries of these species are widely separated in the Amazon (Figs 2, 3 and 6). The greatest distances measured between spawning and nursery areas, as determined by larvae and juvenile presence, were 5,786 km for *B. rousseauxii*, 4,238 km for *B. juruense* and 3,129 km for *B. vaillantii*.

The most extreme migration is undertaken by *B. rousseauxii*, which spawns in the far western Amazon but uses the estuary as its nursery, for a maximum known life history migratory cycle of all size classes of approximately 11,600 km (Figs 5 and 6). The size-class distribution of older juveniles and adults indicates that it takes 1–2 years to reach the Andes during upstream migration from the estuary^9^,^22^,^34^. Based on *B. rousseauxii* size classes recorded from fisheries catches in the western Amazon^19^,^22^ and farther downstream^9^, adults do not return to the estuary but remain in a large area of the western Amazon. The annual migration of adults subsequent to reaching the western Amazon for the first time, however, is probably much shorter; however, based on the distribution of adult size classes, it could still be 1,000–2,000 km or more. Because no other strictly freshwater long-distance fish migrations^7^ close to those discussed in this paper have been reported, *B. rousseauxii* undertakes the longest migration in the world, considerably longer than previously hypothesized^9^. *Brachyplatystoma rousseauxii* migration also surpasses the maximum life cycle migration (6,000 km) reported for anadromous salmon (*Oncorhynchus*)^35^ and is nearly as long as that of the European eel (*Anguilla anguilla* Linnaeus 1758), including the freshwater and marine phases^8^. It is also possible that *B. platynemum* undertake migration from the estuary to the Andean region similar to *B. rousseauxii*, as juveniles of the former are relatively common in artisanal fisheries in the estuary^36^, but much less is known about them^37^.

The general life history patterns of *B. platynemum* and *B. juruense* are similar to those of *B. rousseauxii*, with a more restricted, but still wide, separation of spawning and nursery areas. The more mixed larvae and juvenile size classes of *B. platynemum* across the Amazon indicate that their spawning areas are neither exclusively in the far western Amazon nor are their nurseries restricted to or mostly in the eastern Amazon. In contrast to *B. rousseauxii* and *B. platynemum*, neither adult nor larval *B. juruense* are known in the estuary, and the nursery of the latter species appears to be in the central Amazon. Of the long-distance migratory goliath catfishes, *B. vaillantii* is the only species whose adults and young share the estuary. To date, the maximum distance from the estuary that *B. vaillantii* larvae have been captured is 3,129 km. However, the presence of mature *B. vaillantii* adults in the Marañón River at the Pongo de Manseriche Gorge (4,847 km upstream) and in the Napo of Ecuador (4,754 km upstream) suggests the possibility of spawning in the far western Amazon. Based on commercial catches, *B. vaillantii* is most abundant in the Amazon River mainstem and was rare, if at all present, in Bolivia and Peru above the Madeira Rapids in Brazil. As mentioned above, the new dam bypasses might allow the species to pass the Madeira Rapids, which were previously a barrier^4^.

The presence and abundance of mature goliath catfish in commercial fisheries in the Andean region is a reliable indicator of upstream movement to spawn. The absence of these fish in commercial fisheries during some or all months near the Andes, however, should not be interpreted as direct evidence that they are not present in the region, as fishing activity must also be considered. The striking differences between the monthly relative captures in the Madre de Dios and Ucayali-Urubamba Rivers is most likely due to the absence of fishing in the former rather than the absence of mature fish. The high catches in the Madre de Dios River during the falling river level period during all three years for which data are available should not be interpreted as a greater abundance at this time of year but rather that fishing is possible during these months (Fig. 3). The presence of spawning *B. rousseauxii* and *B. platynemum* in the Madre de Dios River channel during the high water period is corroborated by the relative abundance of their larvae (Fig. 4). The higher density of fish larvae during the rising water period is thus a better indicator of the main reproductive period of goliath catfishes than monthly commercial captures. The best indicator, however, is larval flux, which is the absolute density value of larvae per unit of time in a river section^38^. Drift densities of young fish generally decrease with increased river depth and flow velocity due to the dilution effect caused by higher turbulence in a much greater volume of water^39^. The larval flux index was not used due to the difficulty of obtaining accurate river discharge data near the Andes because of the few hydrological stations that exist. Considering that goliath catfish larvae densities were calculated without discharge calculations and were highest at the beginning of the rainy season and decreased during the January and March peak discharge periods^30^, it is possible that the highest larval flux would be in the latter period if river discharge was considered in the larvae density algorithms.

Fisheries data from the middle Caquetá River in Colombia also indicate that upstream-moving mature goliath catfish are most abundant during the high water period^40^. While upstream areas near the Andes have yet to be investigated as spawning sites in the Caquetá Basin, large *B. rousseauxii* have been reported farther upstream to at least 170 m elevation in the neighboring Putumayo River^23^. In contrast to our data and that of the Caquetá, a gonadal review of 15,000 *B. rousseauxii* specimens captured by commercial fisheries during a 5-year period near Iquitos, Peru at 76 m elevation and approximately 4,000 km upstream of the Amazon River mouth hypothesized that breeding occurs mostly during the low water period and ends at the beginning of rising water^22^. The fisheries in both areas are intensive throughout the year, and fishing effort bias is minimal^22^,^40^. However, those studies analyzed the monthly proportions of mature females by combining maturation stages 3 (advanced maturation) and 4 (ripe)^25^. The uncertainty of the time between stages 3 and 4 raises doubts about combining the two to identify exactly when spawning occurs. It is unknown how far downstream of the Andes or immediate pre-Andean area that long-distance migratory goliath catfish spawn, and the Caquetá and Iquitos data alone do not confirm this. Nevertheless, the presence of maturing populations throughout the year in the Iquitos Region, an area of large rivers and floodplains where prey are more abundant^9^ (Fig. 2, Table 1), suggests that migratory adult goliath fish, especially *B. rousseauxii*, remain in this area to feed and develop their gonads for subsequent reproductive cycles after their first arrival.

A second study in the Iquitos area that included only larval abundance concluded that breeding occurs mostly during receding or low water^41^. Considering that spawning fish are present during all months in the Ucayali headwaters upriver of this site (Fig. 3), it is reasonable to expect the presence of fish larvae downstream near Iquitos during all months. The larval results are based only on abundance without reference to water volume, the latter of which is needed to calculate the absolute abundance index to compare high and low water periods.

A similar but slightly later peak in drifting larval abundance than that of the Madre de Dios River was found for *B. rousseauxii* in the Madeira River, 1,613 km downstream from the former sampling site, with maximum fluxes (larvae or juveniles/second) in January and February and minimal downstream migration in September^38^. Another study using bottom-trawl sampling in the same region of the Madeira River reported different results for *B. rousseauxii*, with relatively high larvae and juvenile abundance (number of fish/haul) during the low water period^42^. However, the study did not consider the bias introduced by the inverse effect of decreased water volume on larval density; thus, seasonal comparisons may not be accurate because the absolute number of larvae may be greater during the high water period but in a much greater volume of water.

As indicated by the upper watershed areas in the Madeira and Ucayali Basins, the goliath catfish spawning zone is located in a mountainous to lowland transition area above about 170 m with relatively high channel slope (declivity = 0.16 m/km) and a lowland downstream area with a much lower declivity (generally less than 0.01 m/km) (Table 1). The river channels in the spawning zone, and in the Andes in general below 300 m, are characterized by gravelly bottoms, as opposed to muddy and soft substrates farther downstream, greater turbidity than downstream, shallower river channel depth (7–20 m versus 40 + m downstream), higher pH (up to 7.9 versus up to 7.1 downstream), higher conductivity (up to 287 microsiemens/cm versus 70 microsiemens/cm downstream), highly saturated O_2_ levels (up to 8.2 mg/l versus up to 6.4 mg/l downstream), and lower average water temperature (26.8 °C versus 28 °C downstream)^20^,^43^. Despite these striking physical differences, there are too few data to support a hypothesis of spawning site selection by long-distance migratory goliath catfishes. Furthermore, limnological and geomorphological data alone cannot be used as explanatory variables because biological factors, such as avoidance of egg and larvae predators, could also play a critical role^9^.

The evolution of long-distance migratory goliath catfish life histories in connection with the western Amazon and western Orinoco, where they also occur, could reflect an ancient evolutionary spawning association with the Andes, as Andean fossils of their genus are known from at least the Miocene 12–11 Ma^16^, although the elevations at that time were probably not much higher than 200 m. The paleo-Amazon-Orinoco in which the ancient catfish species lived flowed north in the low foreland basin of the Andes^44^. *Brachyplatystoma vaillantii, B. rousseauxii*, and *B. platynemum* range widely beyond the Amazon, with all three found throughout the Orinoco Basin and the first two also in the large, short rivers of the Guianas. Juveniles have also been reported in river channels of the Orinoco Basin^45^.

The geographical genetics of three goliath catfish species have been studied, but only *B. platynemum* presented clear population segregation for the Amazon River mainstem and Madeira River^33^, whereas *B. vaillantii* showed no genetic segregation^46^,^47^. Genetic studies of *B. rousseauxii* presented mixed results but indicated a relatively homogenous population in the Amazon Basin, although homing behavior could not be totally excluded^48^,^49^. Regional genetic differentiation of *B. rousseauxii* in the large headwater region of the upper Madera Basin in Bolivia could also indicate homing behavior^49^. The present genetic evidence indicates that long-distance migratory goliath catfish species have few distinct populations in the Amazon Basin, but it does not convincingly eliminate the possibility of homing.

Recent studies have reconstructed the theoretical movement and migration of individuals of the *Brachyplatystoma* species by comparing strontium isotope signatures (^87^Sr/^86^Sr) along transverse sections of their otoliths with the isotope signatures of major river water types^10^,^11^. The main results show that the juvenile phases of *B. rousseauxii* and *B. vaillantii* have strontium isotope signatures of the western Andean tributaries and the Amazon River mainstem, and few present signatures for other water types. The estimated mean size (37 ± 16 mm (mean ± S.D.) of *B. rousseauxii* when it reaches the Amazon River during its downriver drift migration in the Madeira Basin has also been calculated using strontium isotope signatures and the relationship between otolith radius and body length^10^. The length range of *B. rousseauxii* larvae and juveniles captured in the Madeira River in our study varied from 5 to 42 mm (Fig. 5), in agreement with that extrapolated from otoliths. The otolith data corroborate that the nursery habitat of these two species is mostly in turbid rivers, as directly indicated by our study and indirectly indicated by fisheries data^9^. Although considering the eastern Amazon as an obligate region of early goliath catfish life cycles, otolith isotope interpretations also point to the possibility that some of the large eastern clearwater tributaries might be used as nurseries for *B. rousseauxii* and *B. vaillantii*. The eastern estuary (Marajó Bay, Pará River and the lower Tocantins) is heavily influenced by the clearwater Tocantins River, especially during the Amazon River’s low water period^50^; thus, it should be expected that at least part of the estuary goliath catfish nursery population would have a clearwater river otolith strontium signature. Our field studies do not indicate the presence of young (<20 cm) *B. rousseauxii* and *B. vaillantii* in the Xingu or Tapajós Rivers, and they are absent or extremely rare in the fisheries of those areas. A multi-element otolith microchemistry study also indicated that *B. rousseauxii* resided in the Amazon estuary for the first 1.5–2.0 years of life based on Sr:Ca and Ba:Ca ratios^12^.

By presenting the convergence of three lines of evidence—the distribution of mature size classes, downstream migration of larvae/juveniles and otolith signatures—that strongly suggest long-distance goliath catfish migration in the Amazon, this study presents a significant step towards achieving a holistic understanding of the longest freshwater fish migrations in the world and at a mega-basin scale. Furthermore, it sets the hypothetical stage for eventual tagging experiments to understand the exact environmental cues that the fishes use during various life history movements and to test empirically recent homing hypotheses^10^ and at what sub-basin level they occur. The methods used in this paper also raise uncertainties that the present data alone cannot address, such as why the goliath catfishes that use the eastern or central Amazon as nurseries migrate thousands of kilometers upstream to spawn in the far western Amazon associated with the Andes or nearby uplands rather than reproduce much closer in the middle or even lower reaches of the Amazon River mainstem or in the eastern tributaries arising on the Brazilian and Guiana Shields.

Finally, the previous limitations imposed by inferring migration based on the wide separation of nurseries and spawning areas are largely eliminated in this paper by proof of long-distance downstream larvae/juvenile movements in the river channels. Of special relevance is the expected infrastructure development in the Andes, especially the combination of dams, headwater deforestation and mining activity^51^,^52^, which could present major threats to important spawning areas ranging from Colombia in the north to Bolivia in the south. Andean large dams will most likely be much different than those already constructed elsewhere in the Amazon, specifically their high walls. Even if high-wall dams are located upstream of spawning sites, they would greatly alter sediment and nutrient cycles downriver where spawning occurs. The long-distance migratory goliath catfishes provide a profound biological indicator of ecosystem health from the Andes to the freshwater Amazon River plume in the Atlantic, and the impacts on them should be considered in all major infrastructure development.

## Methods

### Definition of Long-Distance Migratory Goliath Catfishes

A modern cladistic classification of the goliath catfish genus *Brachyplatystoma* includes seven species^17^, of which five of the extant six species are easily recognized by commercial fishermen. We consider five (*rousseauxii, vaillantii, platynemum, juruense* and *tigrinum*) to be long-distance (>1,000 km) migratory species. We did not include *B. tigrinum* in this study because it is relatively rare in fisheries, and our data for it were minimal, although small numbers of individuals are commonly present in association with the upstream movement of *B. rousseauxii*. The largest goliath catfishes are *B. filamentosum* and *B. capapretum*, both commonly reaching more than 2.5 m in length and 100 kg in weight. We found no field evidence that they perform long-distance migration (>1,000 km), and their mixed size classes across the Amazon indicate that they spawn widely and in various river types, although shorter migrations are probably involved^12^. They are also the only species that commonly enter floodplain waters^53^, whereas the long-distance migratory species are largely confined to river channels and the estuary.

### Adult Goliath Catfish Distribution and Size Classes

Data for the presence of migratory goliath catfishes, their size classes and maturity stages were obtained by extensive field surveys of local fisheries conducted by the authors since the late 1970 s in six major regions: (1) the Amazon River mainstem from its estuary in Brazil to the confluence of the Ucayali and Marañón Rivers in Peru; (2) major tributary basins (Madeira, Ucayali and Marañón) with headwaters in the Andes; (3) the Purus and Juruá Basins, whose headwaters are associated with the Fitzcarrald Arch, which has a post-Andean uplift origin that produced their low hilly areas, such as the Serra do Divisor^54^; (4) Guiana Shield basins (Negro, Branco and Trombetas); (5) Brazilian Shield basins (Trombetas, Xingu and Tapajós); and (6) the Amazon estuary, including Marajó Bay and offshore fresh waters in the Atlantic. The data from the Andean tributaries in Colombia and Ecuador were obtained from the literature. Published goliath catfish data, including fisheries catches, maturity stages and size classes, were especially important for the Caquetá and Putumayo basins, which have been investigated by Colombian scientists^19^,^23^. Photographic proof and coordinates of goliath catfishes for the Napo Basin near the Andes was provided in a recent thesis^24^.

The presence of the migratory goliath catfishes and their maturity stages were verified by the authors from specimens in urban markets and complemented by local interviews. The maturation stages of females and males were based on macroscopic gonadal characteristics: ripe, ready-to-spawn individuals, spent or very recently spent^25^. Wherever present, goliath catfishes are known to the local people, and nearly all oral reports we received were eventually verified by actual specimens either from fishers or our own observations. Mature fish were considered *abundant* in a fishing area when they were among the 20 most important food species, *frequent* when they regularly appeared in markets in small quantities, and *rare* when observed by the authors but their presence was not well known by local fishers.

Andean surveys based on interviews to establish the maximum elevations that goliath catfishes reach were conducted in the Amazon River’s two largest Andes-Amazon sub-basins, the Madeira and Ucayali. For the Madeira Basin, the Madre de Dios sub-basin in Peru was surveyed from the city of Puerto Maldonado in the lowlands (<200 m) to tributaries 4,000 m upstream, including the Manu, Alto Madre de Dios and Inambari Rivers, whose headwaters rise in the high Andes. Surveys in the Ucayali Basin ranged from near Cusco at 3,300 m to Atalaya at 200 m and included the Vilcanota, Urubabamba, Tambo and Ucayali Rivers. No evidence of long-distance migratory goliath catfishes was found above 250 m elevation in the Andes (Table 1).

Two large datasets of unpublished goliath catfish fork length and gonadal maturity stages are reported here for the first time: Madre de Dios River commercial fisheries based in the city of Puerto Maldonado between April 2002 and April 2004 in the Upper Madeira Basin of Peru and Urubamba and Ucayali commercial fisheries between July 2004 and August 2005 based in the city of Atalaya (Table 2). The Madre de Dios fisheries are within 150 km of the Andes, and those of Atalaya are at the edge of the Andes.

### Ichthyoplankton Data

Given the lack of tagging experiments to detect upstream movements in the days or weeks before spawning, a more substantial understanding of goliath catfish reproductive periods in the western Amazon is provided by the seasonal downstream drift of their larvae, which indicates when spawning occurs. We define larvae as the fish phase between hatching to complete loss of embryonic and larval organs^55^, with the juvenile stage beginning at approximately 20 mm^56^. Fish larvae larger than 2.7 mm were measured, and they were identified by the number of caudal and pre-caudal myomeres^56^. Year-round larvae and juvenile collections were conducted in the Madeira, the Amazon’s largest sub-basin, and these results were compared to all the data available for the Amazon, including all specimens captured in various years, seasons and locations. For the Madeira Basin, the Madre de Dios River in southeastern Peru was selected as the site near the Andes to test for the presence of headwater larvae because commercial fisheries based in the city of Puerto Maldonado revealed the at least seasonal presence of mature goliath catfishes near the Andes^20^.

Total length and abundance data of goliath catfish larvae and juveniles were investigated near the Andes in the Madre de Dios, Ucayali and Urubamba Rivers; in the lowlands in the large channels of the Madeira and Amazon rivers; and in the freshwater areas of the Amazon River estuary. Samples were collected during daylight hours with ichthyoplankton nets with a mesh size range of 300–500 μm and trawl nets with a mesh size of 5 mm^30^,^56^,^57^. The fish larvae collection method was approved by the Brazilian Institute of the Environment - IBAMA (SISBIO- permission number 4419) in Brazil and the Dirección General de Extracción y Producción Pesquera para Consumo Humano Directo from the Ministry of Production in Peru, with in-country legal deposit protocols of the Museo de Historia Natural, Lima.

Standardized larval density data^30^ were collected weekly using ichthyoplankton nets in the Madre de Dios River between November 2004 and August 2005 (Fig. 4). The sampling was conducted at five points across five cross-channel transects along 12 km of the river. Two samples were taken at each site: near the surface (1 m depth) and near the bottom (70% of the maximum depth). A mechanical flowmeter (General Oceanics 2030 R) was installed at the mouth of the net to estimate the volume of water sampled. The monthly drifting larvae densities were estimated based on the average of all samples.

### River Distance Calculations

The distances from the Amazon River mouth to the studied areas were determined using the Barrier Analysis Tool (BAT) as an extension of ArcMap 10.2, which was developed for The Nature Conservancy (Software Developer: Duncan Hornby of the University of Southampton’s GeoData Institute). The tool uses point data to divide a routed river network (polylines with node to node coding) into connected networks from which a direct path distance calculation can be made.

To test the long-distance downstream larvae and juvenile migration hypothesis, the size of larvae and juveniles in the ichthyoplankton samples from the Madre de Dios, Madeira and Amazon Rivers and the estuary were related to the distance of the sampling points to the Amazon River mouth by the exponential equation *L* = a × *e*^b×*D*^, where *L* is the size in millimeters and *D* is the distance in kilometers from the Amazon River mouth. An exponential model was chosen because it had biological significance and provided the best data fit.

### Climate Data

Hydrological data were not available for most of the Andean areas we surveyed; thus, we used local precipitation data as a proxy for river level seasonality. Precipitation data were supplied by the Servicio Nacional de Meteorología e Hidrología del Peru (SENAMHI). Because there were no precipitation records for Puerto Maldonado (Madre de Dios) in November 2004, we used the average November precipitation registered in a historical series of 43 years.

### Note

Herewith the authors declare that the study (manuscript no: SREP1622389T) was completed in accordance with the laws of Brazil and Peru and the fish larvae collection method was approved by the Brazilian Institute of the Environment - IBAMA (SISBIO- permission number 4419) in Brazil and the Dirección General de Extracción y Producción Pesquera para Consumo Humano Directo from the Ministry of Production in Peru, with in-country legal deposit protocols of the Museo de Historia Natural, Lima.

## Additional Information

**How to cite this article**: Barthem, R. B. *et al*. Goliath catfish spawning in the far western Amazon confirmed by the distribution of mature adults, drifting larvae and migrating juveniles. *Sci. Rep.* **7**, 41784; doi: 10.1038/ srep41784 (2017).

**Publisher's note:** Springer Nature remains neutral with regard to jurisdictional claims in published maps and institutional affiliations.

## Figures and Tables

**Figure 1:**
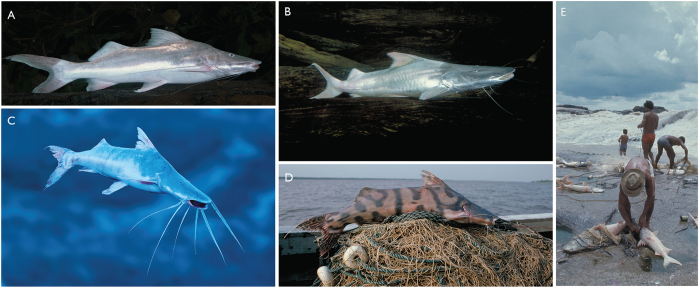
Migratory goliath catfishes (*Brachyplatystoma*, Pimelodidae). (**A**) *B. vaillantii* (piramutaba in Portuguese, pirabutón in Spanish); (**B**) *B. rousseauxii* (dourada in Portuguese, dorado in Spanish); (**C**) *B. platynemum* (babão in Portuguese, mota flemosa in Spanish); (**D**) *B. juruense* (zebra in Portuguese, zebra in Spanish); (**E**) Dorado migrations exploited by fishermen. The Santo Antônio Dam on the Madeira River now drowns the Teotônio Rapids (shown here) where *B. rousseauxii* (species in photo) and *B. platynemum* were previously exploited and easily detected when migrating. Photos by M. Goulding.

**Table 1:** Summary of goliath catfish data locations where mature goliath catfishes were captured in major rivers in Brazil, Bolivia and Peru by the authors, complemented by documented data from studies in Colombia and Ecuador: L1^19^, L2^23^, L3^22^, and L4^24^.

Locality	River	Distance from the Amazon River mouth (km)	Elevation (m)	Data Source	Br	Bv	Bp	Bj
Tefé	Solimões	2,421	30	O		R		
Porto Velho	Madeira	2,852	46	O	R			
Boa Vista	Branco	2,618	55	O	R			
Leticia	Solimões-Amazonas	3,493	55	O	A		A	
Iquitos	Amazonas	3,978	76	L3	A			
Araracuara	Caquetá-Japurá	3,755	115	L1	A	F	F	A
Curanja	Purus	3,834	140	O	F			
Fishing area	Juruá	5,039	157	I	F			
Puerto Maldonado	Madre de Dios	4,035	169	O	A		A	
Puerto Leguizamo	Putumayo-Içá	4,645	170	L1, L2	F		A	A
Atalaya	Ucayali-Urubamba	5,788	198	O	A		A	A
Puerto Grether	Ichilo	4,918	231	O	F		F	
Fishing area	Napo	4,754	236	L4	F	F	F	F
Pongo de Manseriche	Marañón	4,847	246	O	F	F	F	F
Rurrenabaque	Beni	4,324	250	O	A			

Types of field data include direct observation by the authors (O) and interviews of fishermen by the authors (I). The presence of mature individuals of *Brachyplatystoma rousseauxii* (Br), *B. vaillantii* (Bv), *B. platynemum* (Bp) and *B. juruense* (Bj) was ranked as Abundant (A), Frequent (F) or Rare (R).

**Table 2:** Ready-to-spawn or recently spawned goliath catfish (*Brachyplatystoma rousseauxii*) near the Andes.

Species	Sex	Maturity Length		Number of Measured fish	
		Fork Length (cm)		Madeira headwaters	Ucayali headwaters
		Min	Max	Madre de Dios	Ucayali and Urubamba Rivers
*B. rousseauxii*	Female	67.7	137	400	2,087
	Male	65.8	122	296	2,565
*B. juruense*	Female	45.1	73.1		380
	Male	43.3	64.7		148
*B. platynemum*	Female	50.2	89.0	1,200	339
	Male	43.7	82.0	1,277	169

Only fully mature goliath catfishes are found in the far western Amazon.
